# Oyster Larvae Settle in Response to Habitat-Associated Underwater Sounds

**DOI:** 10.1371/journal.pone.0079337

**Published:** 2013-10-30

**Authors:** Ashlee Lillis, David B. Eggleston, DelWayne R. Bohnenstiehl

**Affiliations:** Department of Marine, Earth & Atmospheric Sciences, North Carolina State University, Raleigh, North Carolina, United States of America; National Institute of Water & Atmospheric Research, New Zealand

## Abstract

Following a planktonic dispersal period of days to months, the larvae of benthic marine organisms must locate suitable seafloor habitat in which to settle and metamorphose. For animals that are sessile or sedentary as adults, settlement onto substrates that are adequate for survival and reproduction is particularly critical, yet represents a challenge since patchily distributed settlement sites may be difficult to find along a coast or within an estuary. Recent studies have demonstrated that the underwater soundscape, the distinct sounds that emanate from habitats and contain information about their biological and physical characteristics, may serve as broad-scale environmental cue for marine larvae to find satisfactory settlement sites. Here, we contrast the acoustic characteristics of oyster reef and off-reef soft bottoms, and investigate the effect of habitat-associated estuarine sound on the settlement patterns of an economically and ecologically important reef-building bivalve, the Eastern oyster (*Crassostrea virginica*). Subtidal oyster reefs in coastal North Carolina, USA show distinct acoustic signatures compared to adjacent off-reef soft bottom habitats, characterized by consistently higher levels of sound in the 1.5–20 kHz range. Manipulative laboratory playback experiments found increased settlement in larval oyster cultures exposed to oyster reef sound compared to unstructured soft bottom sound or no sound treatments. In field experiments, ambient reef sound produced higher levels of oyster settlement in larval cultures than did off-reef sound treatments. The results suggest that oyster larvae have the ability to respond to sounds indicative of optimal settlement sites, and this is the first evidence that habitat-related differences in estuarine sounds influence the settlement of a mollusk. Habitat-specific sound characteristics may represent an important settlement and habitat selection cue for estuarine invertebrates and could play a role in driving settlement and recruitment patterns in marine communities.

## Introduction

Most bottom-dwelling marine organisms have a biphasic life cycle, consisting of a dispersing larval phase that develops in the plankton prior to settlement into seafloor habitat and a sessile or sedentary adult phase. Successful recruitment of marine larvae, beginning with larval settlement into quality habitat, is fundamental to replenishing populations and structuring benthic communities [1]. Central to understanding marine population and community dynamics is knowledge of the physical, behavioral and physiological processes that influence variation in the arrival and settlement of planktonic larvae into suitable nursery habitats [1]–[5]. Larval responses to environmental cues, including salinity, depth, turbulence and chemical compounds, can have a substantial influence on their dispersal trajectory and subsequent settlement location [3], [4], [6]. Orientation, habitat selection and settlement in response to physical and chemical stimuli have been demonstrated for larvae of a variety of taxonomic groups, and larvae likely integrate a suite of sensory cues at multiple spatial scales to successfully locate preferred settlement substrates [3], [7], [8]. Studies of larval invertebrate settlement cues have focused largely on chemical compounds [9], [10] and habitat characteristics, such as surface texture or near-bottom hydrodynamics [11]–[13], but these stimuli are only detectable by larvae that are in close proximity of the seafloor [7], [12]. While a combination of habitat characteristics, such as local flow patterns, chemical exudates, light intensity and substrate topography are used by benthic invertebrate larvae in small-scale habitat selection, it is still unclear how larvae effectively locate discrete patches of bottom settlement habitat as they disperse over kilometers, especially for species that are relatively weak swimmers compared to surrounding currents.

Underwater sounds produced by physical and biological processes are increasingly recognized as a potentially effective signal for larvae of benthic organisms to locate patchily distributed—yet acoustically distinctive—settlement habitat, such as coral reefs and other subtidal habitats [14]–[17]. For example, biological sounds produced by conspecifics, prey-species, or habitat-forming species dominate the underwater soundscape of certain benthic habitats [14], [15], [18] and could reliably indicate proximity to appropriate settlement sites. Compared to other sensory stimuli, such as light and chemical compounds, sound is unique in having relatively long-range transmission, a presence at all depths, and propagation that is independent of currents [19], [20]. Field experiments of replayed reef sounds have demonstrated sound-enhanced settlement rates in fish and crustacean larvae from coral reefs and rocky habitats, and provide convincing evidence that these animals use acoustic cues in orientation and settlement [17], [21]–[23]. Coral reef fish were attracted to specific frequencies of reef noise signals [24] and exposure to the sounds of settlement habitats, but not to the sounds of other habitats. Specific sound frequencies also affected larval behavior, settlement, and metamorphosis of crab larvae in lab experiments [16], [25]. Many taxa of invertebrates possess mechanosensory structures capable of serving as sound receptors [26]–[30], and there is growing evidence of a role for underwater sound in the behavior and settlement of larval echinoderms, cnidarians and mollusks [30]–[32]. Larvae of a coral species were shown to move toward reef sounds in playback experiments [33], and vessel noise was found to induce larval settlement in the New Zealand green-lipped mussel [32].

The majority of habitat-associated soundscape characterization has occurred in a single region in New Zealand [15], [34], and ambient sound as a settlement cue has been studied primarily for a handful of rocky reef and coral reef species in the Southern hemisphere. Compared to the knowledge of tropical coral reef soundscape characteristics and their influence on larval stages of reef species [21], [23], [24], the habitat-related soundscape of most estuarine and coastal systems and the role of these sounds in larval processes is unknown. Estuaries and coasts in sub-tropical and temperate regions represent vast areas of critical nursery and adult habitat for a multitude of commercially and ecologically important species. Though little studied, the estuarine soundscape is likely to have considerable sonic variation related to the discrete habitats and associated communities present, such as seagrass, salt-marsh, oyster reef, and unstructured soft (sand or mud) bottoms.

Here, we use the oyster reef soundscape as a novel study system to examine the possibility that habitat-related sound characteristics influence larval settlement processes. Oyster reef soundscapes are of particular interest as a larval cue because reefs are patchily-distributed and productive habitats that harbor many sound-producing organisms (e.g. sciaenid fish, snapping shrimp) [35], [36], and provide structure for a multitude of obligate reef-dwellers with dispersing larvae [36], [37]. Inhabitants of an oyster reef create sound during their activities (e.g. feeding, reproduction, courtship, movement, defense), and the relatively high density of soniferous organisms, along with the physical structure of the reef itself, will produce a higher level of sound with distinct characteristics compared to less densely populated and differently structured environments, such as soft sediment bottoms. In addition to species that purposefully produce sound, animal activity, such as crushing of shelled prey or burrow excavation, is likely to contribute substantially to the unique soundscape in a densely populated reef habitat.

While larvae of certain bivalves can respond to acoustic stimuli [31], [32], habitat-associated underwater sound as a settlement cue has not previously been investigated for a bivalve or estuarine species. Moreover, the potential for an estuarine soundscape, particularly oyster reef sound, to act as a larval settlement signal has not been considered. The overall aim of this study was to compare the acoustic characteristics of two common estuarine habitat-types (oyster reef and off-reef soft bottoms) and to investigate the role of soundscape cues in the settlement of a reef-forming larval bivalve, *Crassostrea virginica*, to determine if habitat-related sound affects settlement.

## Methods

To determine whether the estuarine habitats of interest differed in their acoustic spectral characteristics, and therefore represented a potential settlement cue, field recordings of ambient sound on oyster reef and nearby soft-bottom habitats were conducted several times during the peak oyster settlement months. Using laboratory and field experimental approaches, we then examined the settlement response of oyster larvae to natural ambient sound associated with suitable (oyster reef) and unsuitable settlement habitat (unstructured soft-bottom) to test the hypothesis that oyster larval settlement would be higher in the presence of sound associated with their preferred settlement habitat compared to sounds of other habitats. In laboratory experiments, we quantified oyster larval settlement in cultures exposed to two estuarine soundscapes (oyster reef vs. unstructured soft bottom), as well as a no sound control. Because acoustic stimuli matching field conditions are particularly difficult to produce in small laboratory tanks [38], [39], we conducted a field experiment to test if oyster larval settlement was higher in larval “houses” anchored above oyster reefs compared to above unstructured soft bottom. Permission to conduct field work in Pamlico Sound waters and the West Bay oyster reserve was granted by the North Carolina Division of Marine Fisheries permit numbers 708396 and 1012889.

### Estuarine soundscape measurements

**Figure 1:**
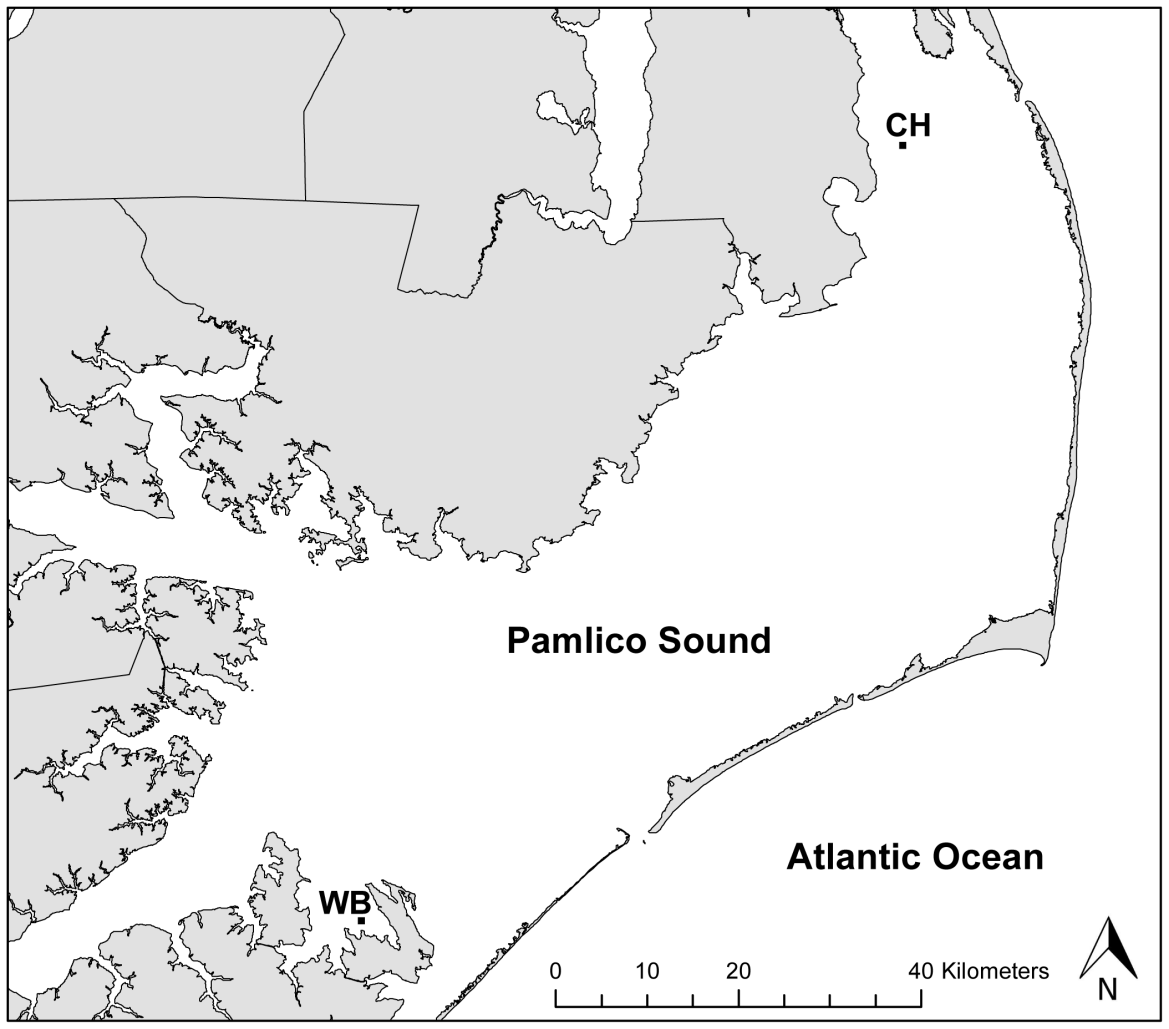
Map of Pamlico Sound, NC. Oyster reserves at which sound recordings were made are marked (CH  =  Crab Hole; WB  =  West Bay). Field-based settlement experiment was conducted in West Bay.

During 2010, oyster reefs and nearby soft-bottoms (∼ 2 km from oyster reefs) at two sites in Pamlico Sound, North Carolina (Fig. 1) were acoustically sampled simultaneously over dusk and nighttime periods during new moon (± 3 days) periods in July, August and September to quantify differences in habitat-related sound. West Bay and Crab Hole are two oyster reserves within Pamlico Sound that form part of a network of no-take reserves throughout the estuary established by the North Carolina Division of Marine Fisheries in 1996. These two reserve sites are separated by 100 km and were selected because they represent a range of variability in oyster reef size and structure in our system – West Bay reef is relatively small (8093 m^2^) and sheltered compared to the larger (55 400 m^2^) and more exposed Crab Hole reef. Continuous recordings of ambient habitat sound were collected using underwater recording systems consisting of a SQ26-08 Sensor Technology omnidirectional hydrophone, with an effective sensitivity of −169±1 dB re 1V/μPa over the 0.1–28 kHz frequency range. The hydrophone was positioned 1 meter from the seafloor and connected to an M-Audio Microtrack II digital recorder (sample rate: 48 kHz) in a waterproof housing at the surface. For reef recordings, hydrophones were stationed within the reserve boundary but on unstructured bottom approximately 25 meters from reef structure.

Digital recording samples were analyzed in MATLAB using purpose-written code. Spectrograms were first inspected for anomalous transient or anthropogenic noise and acoustic power spectra for concurrent recordings were produced to make comparisons of the acoustic characteristics of the two habitat types.

### Source and maintenance of oyster pediveligers

Prior to each laboratory- or field-based settlement trial, eyed *Crassostrea virginica* pediveligers (i.e., competent to settle) were dry-shipped on ice overnight from the Horn Point Laboratory (HPL) oyster hatchery (University of Maryland) where broodstock was obtained from Chesapeake Bay oyster populations. Following arrival to the laboratory in North Carolina, USA (Raleigh for lab experiments and Morehead City for field experiments), larvae were warmed in dishes of 10 µm filtered seawater at a salinity matching HPL's rearing conditions (10–15 psu), and held at room temperature (23–25°C) for the duration of the experiments. Twice in 2011, larvae were available from a local NC hatchery (Bear Creek Shellfish Hatchery), and were obtained for use in a laboratory trial and a field trial, following the same protocol as other trials.

### Larval settlement experiments (Laboratory)

#### Sound treatments

The field soundscape sampling effort described above generated a library of 4-to-5-hour-long recordings of reef and off-reef sounds from which experimental playback treatments were selected. Sound treatments used in laboratory larval settlement experiments consisted of replaying the habitat sounds recorded *in situ* at oyster reef and adjacent off-reef soft bottom habitats at West Bay and Crab Hole sites. Prior to use in experiments, waveforms and spectrograms of the candidate recordings were visually inspected using Audacity™ software to ensure the absence of anthropogenic or anomalous noise.

In the four trials comprising the first laboratory settlement experiment in September 2010, a 15-minute recording of dusk-time oyster reef sound was played continuously as the sound treatment. The 15-minute reef sound-clip was selected from a September recording of the West Bay oyster reserve on a new moon, contained no anthropogenic noise, and was typical of reef sounds recorded within Pamlico Sound reef habitats. For the second set of laboratory experiments in July and August 2011, in which both reef and off-reef sound treatments were applied, pairs of simultaneously recorded audio clips were used in each trial. The recordings used in the five trials of experiment 2a were 15-minute samples from recordings made at the West Bay oyster reef and off-reef sites at dusk in July 2010. To avoid concerns of pseudoreplication associated with the use of a single recording in experimental playback experiments [40], several candidate recordings were selected from multiple oyster reserves for use in experiment 2b. Three 15-minute July recordings, two from the West Bay reserve and one from the Crab Hole reserve, were each used in three of the 9 trials. Recordings from these times were selected for treatments to match the timing of each lab experiment and also because oyster settlement typically occurs between mid-June to late-September in the Southeastern United States, with peaks often occurring in early July and September [35].

Prior to the start of experimental trials, in-tank recordings using a calibrated omni-directional hydrophone (Sensor Technology SQ26-08) and digital recorder (M-Audio Microtrack II) were used to adjust the speaker levels in sound treatments to reflect typical oyster reef or off-reef sound pressure levels, and to the degree possible, match the acoustic spectrum of the original recordings. For these calibrations, the hydrophone was placed just below the water surface where the experimental jars used in trials were located. Hydrophone recording in the no sound (control) tanks also confirmed the absence of substantial noise from these treatments. Additional measurements of particle motion were made after the experiments were concluded to ensure that the total acceleration during the playbacks approximated far-field conditions. Particle acceleration measurements were made following the methods of Glade [41] and others [19], [42], [43], using the pressure gradient measured between two closely space Brüel and Kjaer (B&K) 8103 miniature hydrophones (sensitivity of ± 1 dB re 1V/μPa over the 0.1 Hz to 20 kHz frequency range). The hydrophones were mounted with a 2.0 cm vertical separation near the top of the tank and recorded digitally with a single B&K LAN-XI Notar system. At high frequencies, as the acoustic wavelength becomes small compared to the hydrophone separation, this method of measuring sound intensity becomes increasingly biased [41]. We therefore limited the frequency bandwidth of our acceleration measurements to the range of 0.1–6 kHz.

#### Experiment 1

Initial trials to test the effect of underwater sound on oyster larval settlement occurred in September 2010. Oyster settlement (the proportion of oyster larvae settled at the end of a trial) was measured for groups of larvae randomly assigned to tanks with or without playback of continuous oyster reef sound. During this preliminary experiment, a series of four trials were conducted, each lasting between 2–5 days as part of method refinement to determine the most appropriate trial length to ensure measurable settlement rates. Trials were conducted in complete darkness to remove any effect of light on settlement behavior, as experimental light conditions have been implicated in producing contradictory results in studies of oyster settlement response to physical factors [35]. Dark conditions were also intended to encourage settlement and reduce trial length, since higher settlement rates have been found in dark or low light conditions for *Crassostrea virginica* [44], [45].

**Figure 2:**
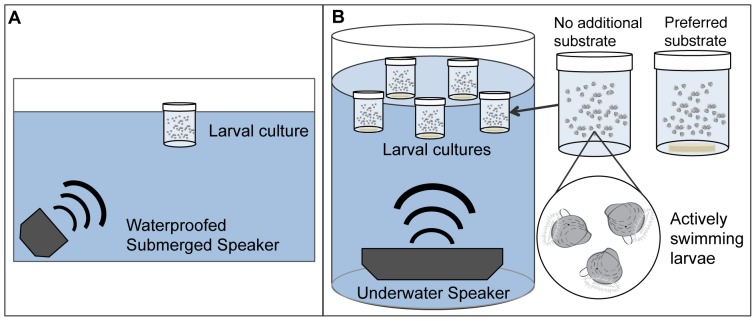
Larval settlement experiment (Laboratory) setup. a) Side view schematic diagram of a replicate experimental tank (0.25 m×0.5 m) used in lab-based settlement experiment 1, showing the placement of a submerged speaker and container housing oyster larvae within a water bath, and b) Cylindrical treatment tank (20-L, 0.3 m water depth) used in lab experiment 2 containing an underwater speaker and five larval culture jars containing groups of actively swimming larvae. Larger view of larval culture jars with and without substrate is shown. No additional substrate was provided in experiment 1 and 2a; oyster shell discs were placed in the bottom of containers in experiment 2b.

At the beginning of each trial, groups of 100 actively swimming pediveliger larvae were randomly assigned to a 20-L glass experimental tank where they were housed 0.2 m off-bottom in a 100 mL plastic container (1.5 mm clear polystyrene, Sterilin® 185BP) filled with 10 µm filtered seawater (Fig. 2a). Each tank also contained a submerged speaker (Altec Lansing BXR1220) held in a watertight plastic bag. Tanks were randomly designated as reef sound or no sound (control) treatments, and each trial consisted of 3–5 replicate tanks depending on the amount of available larvae and audio equipment. Sound treatments continuously played a recording of ambient oyster reef sound from a laptop media player for the duration of a trial. At the end of each trial, larval containers were removed from experimental tanks and examined under a dissecting microscope to count the number of settled (attached to sides or base of the container) and unsettled (swimming or crawling) oyster larvae. A pipette was used to gently agitate larvae to confirm whether they were attached to the container surface. The response variable was proportional larval settlement in each replicate. An ANOVA was used to test for a sound treatment effect, with trial as a fixed factor and a trial by treatment interaction term. For all analyses, assumptions of normality and homoscedasticity were verified by inspection of plotted residuals versus fitted values and a Levene's test of equal variances, respectively.

#### Experiment 2

A second set of experiments was conducted in 2011 to expand upon the scope of the preliminary experiment by including an additional off-reef sound treatment, increasing replication, and improving the quality of laboratory sound playbacks through the use of cylindrical tanks and underwater speakers. Three experimental arenas were constructed, each consisting of a 20-L cylindrical water bath with an underwater speaker (Clark Synthesis Aquasonic AQ339 Underwater Loudspeakers, frequency range: 20 Hz–17 kHz) placed on the bottom 0.2 m below larval culture containers (Fig. 2b). Speakers were connected to a laptop with an audio player. Soundproofing foam was used around and beneath each tank to reduce sound transfer. Using this setup, the effect of distinct habitat sounds (reef vs. off-reef sound) was compared in a set of laboratory larval settlement experiments conducted with three treatment levels (no sound, reef sound and off-reef sound) using a randomized block design with trial as the blocking factor. A total of 14 trials were conducted, 5 trials using larval containers with no added substrate (Experiment 2a) and 9 trials wherein larvae were provided a 3-cm diameter oyster shell disc as settlement substrate (Experiment 2b). All trials were conducted under complete darkness for 48-hour periods.

For each trial, 15 groups of 100 actively swimming pediveliger larvae were placed in 80-mL containers and randomly assigned to an experimental tank (5 larval cultures per tank). The experimental tanks were randomly designated as reef sound, off-reef sound or no sound treatments prior to each trial, and for sound treatments a recording played continuously for the duration of the trial. As in experiment 1, oyster settlement at the conclusion of a trial was measured in each container under a dissecting microscope as the proportion of individuals attached to the substrate or container surfaces, and the mean proportional settlement for each replicate was calculated. For each of the two experiments, randomized block ANOVAs were used to test for a difference in the response variable (mean settlement) amongst sound treatments, blocked by trial. Significant differences in mean settlement among the sound treatments were identified with a Tukey's Honestly Significant Differences test.

### Larval settlement experiments (Field)

**Figure 3:**
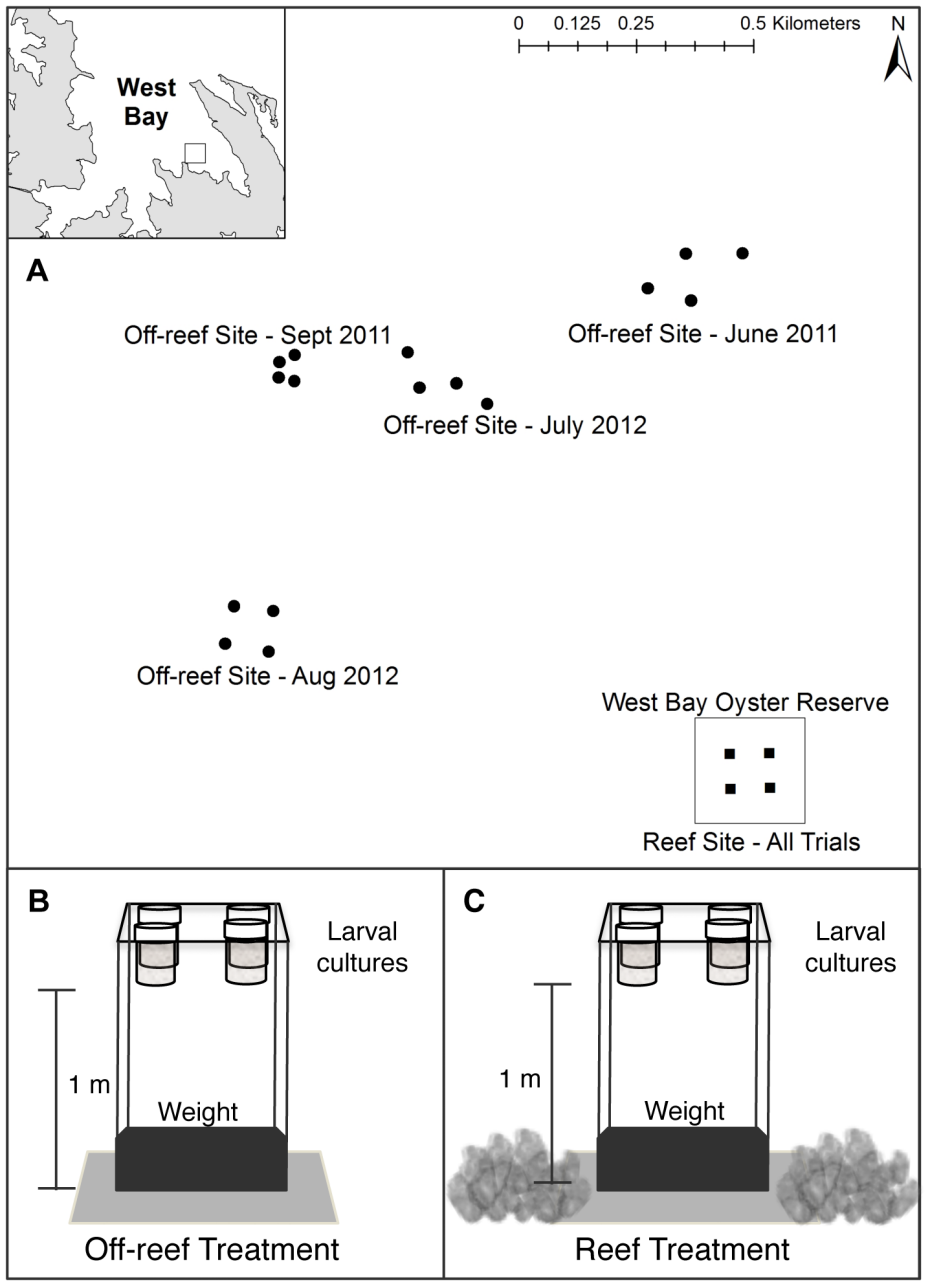
Larval settlement experiment (Field) setup. a) Map of field experiment site in West Bay. Circles denote locations of “off-reef” replicates, and squares are “reef” replicates. The extent of the West Bay oyster reserve is indicated by the box bordering the reef replicates. Inset shows experiment location in West Bay, b) Schematic of the larval housing placed at each replicate in off-reef sites and c) in the reef site. Larval cultures were suspended at 1 m above the seabed at each location, with the two habitats providing the ambient sound treatments.

To further overcome limitations related to the reproduction of sound using speakers in small tank experiments [14], [38], a field-based settlement experiment was conducted on oyster reef and adjacent off-reef habitats that served as ambient sound sources for cultures of hatchery-reared oyster larvae. Four trials were completed in June and September of 2011 and July and August of 2012. An oyster reserve in West Bay, Pamlico Sound, North Carolina served as the “reef sound” site and the “off-reef sound” site was a soft sediment bottom located ∼ 800–1000 m from the reserve (Fig. 3a). Off-reef sites were also selected to match the reef site depth of approximately 3 meters.

In each of four trials, four replicate “larval housings” were placed at each site. Larval housings consisted of sample jars identical to those used in laboratory trials and each jar contained an oyster shell disc as settlement substrate. The number of larval cultures used in each replicate larval housing varied among trials (1–4 jars per replicate) based on availability of larvae and logistical constraints. As in laboratory settlement experiments, groups of 100 larvae were placed in the tightly sealed jars prior to deployment at the habitat sites, thereby exposing larvae to habitat sounds while excluding other potential habitat-associated cues such as differences in water chemistry. Larval housings were suspended 1 meter above the seabed (Fig. 3b, 3c). At the reef site, larval housings were deployed within the reserve boundaries, but on sand bottom adjacent to oyster reef structure to minimize potential differences in light or visual cues between the reef and off-reef habitats. Each experimental trial lasted 48–72 hours, determined by field site deployment and retrieval logistics during a given trial period. After a trial, larval housings were retrieved via scuba divers, and settlement discs preserved in 95% ethanol and transported to the laboratory to measure settlement as the proportion of larvae in a culture. The response variable, mean proportional settlement, was calculated for each replicate. An ANOVA model was used to test for differences in mean proportional settlement between the habitat treatments, with trial as a fixed factor and a trial by treatment interaction term.

The different soundscapes of oyster reef and off-reef sites at West Bay were well-characterized prior to this field experiment, providing support for the assumption that the sites could provide distinct sound treatments for the experimental units (see soundscape measurement results below). Recording equipment to monitor the site acoustics during field experiment trials was unavailable until the final trial in August 2012, for which a long-term acoustic recorder (DSG-Ocean, Loggerhead Instruments) was deployed at each site on a recording schedule of 1 minute every 15 minutes at a sampling rate of 50 kHz. Each DSG consisted of an individually calibrated HTI-96-MIN hydrophone (Hi-Tech Inc), with the frequency-amplitude response of the system being flat (+/− 0.2 dB) over the range of ∼ 100–25,000 Hz.

## Results

### Estuarine soundscape measurements

**Figure 4:**
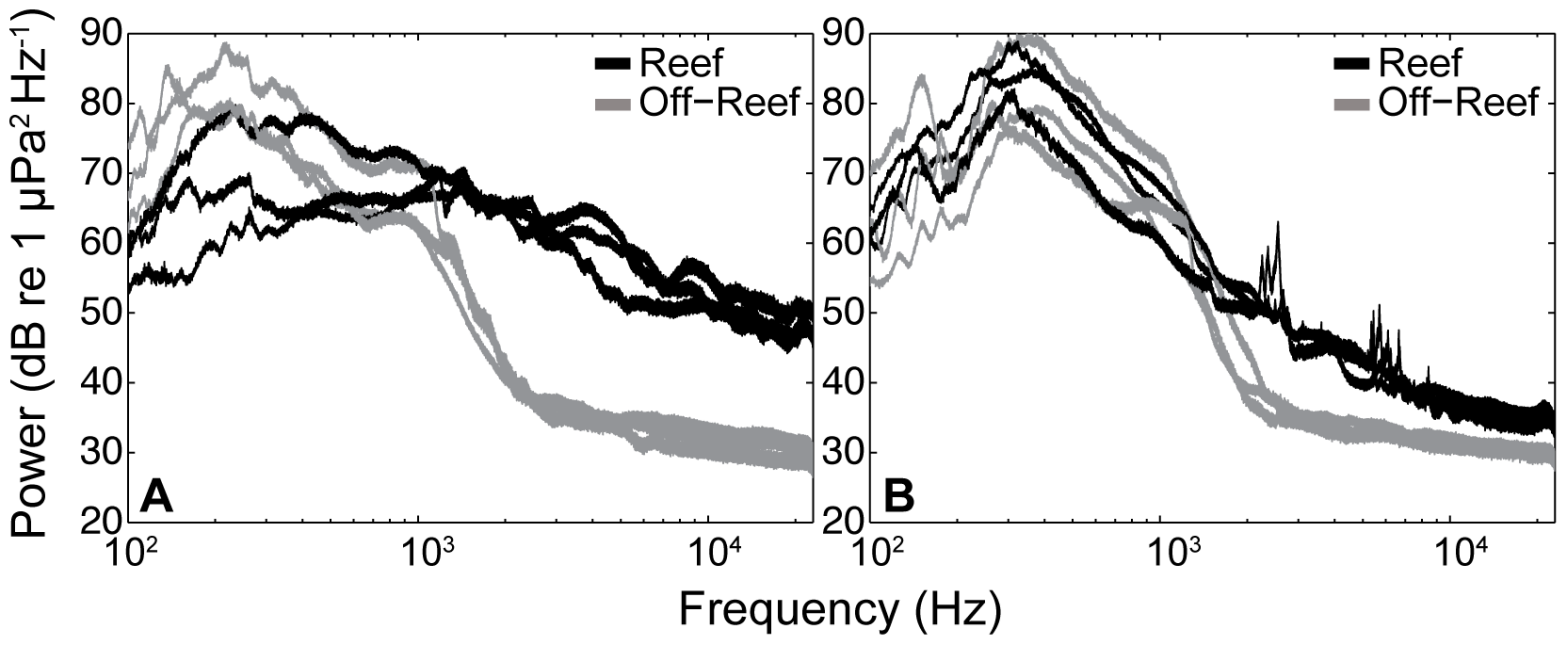
Oyster reef and off-reef acoustic spectra for two Pamlico Sound sites. Power spectral density for sounds recorded on and off-reefs taken monthly July-September at (a) West Bay and (b) Crab Hole. Because the field recordings differed in total length, each multi-hour recording was shortened to a one-hour sample that began at sunset.This produced six pairs of recordings collected simultaneously in each habitat type. The displayed data represent the median spectra for each hour-long sample calculated from a series of non-overlapping 10-sec duration windows.

The spectral composition of oyster reef sound was consistently different compared to nearby soft bottom habitats for the July, August and September sampling periods at the two field sites (Fig. 4). The difference between habitat types was more distinct at the West Bay site than the Crab Hole site (Fig. 4a), but both sites showed consistently higher levels of acoustic energy in the 1.5–20 kHz range at reefs compared to the simultaneous recordings made at off-reef locations. The acoustic spectra suggest that acoustic characteristics of the estuarine soundscape can vary over relatively small spatial scales (kilometers) related to habitat structure. The oyster reef recordings consisted primarily of the high energy broadband “snaps” produced by snapping shrimp (*Alpheus heterochaelis*) [46], as well as sounds in the 150–1500 Hz frequency range associated with the calls of common sciaenid species living within reefs such as oyster toadfish, weakfish, croaker and spotted seatrout [47], [48]. Off-reef recordings were characterized by the relatively lower frequency (100–800 Hz) sounds associated with abiotic sources such as wind and waves, as well as 100–1500 Hz sounds associated with the vocalizations of drumming fish aggregations [47], and a low level of sound in the higher frequency range typically derived from invertebrate sources [24], [46].

### Larval settlement experiments (Laboratory)

#### Sound treatments

**Figure 5:**
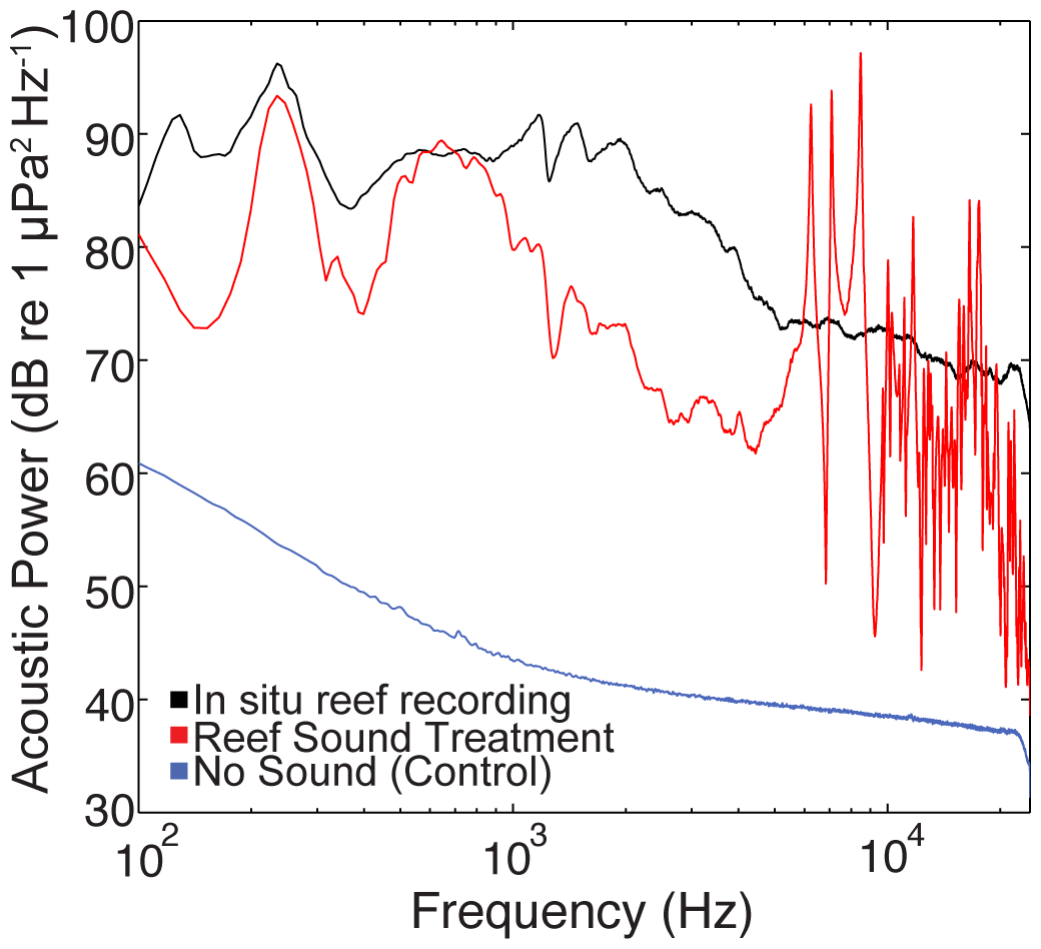
Spectral composition of sound in experiment 1 tanks (reef sound and no sound (control) treatments). The black line represents the spectrum of the original in situ recording from West Bay oyster reserve that was replayed in reef sound tanks. Power spectral density estimated via Welch's method (Hamming window, 1-sec averages with 50% overlap).

The conventional computer speakers placed in waterproof bags and used in laboratory experiment 1 to replay oyster reef sounds produced a sound spectrum that generally matched the sound pattern of the original field recordings; however, there was a somewhat limited ability to reproduce all frequencies consistently (Fig. 5). In particular, there was a reduction in sound level in the 300–500 Hz and 2–5 kHz range. Nevertheless, the reef sound treatment did capture components of the higher frequency range of the original recordings that were associated with high frequency pulses of snapping shrimp sound, which provided a distinct sound treatment for the oyster larval cultures in laboratory tanks. The mean sound level produced in treatment tanks was 121.8 dB re 1 µPa, which was reasonably consistent with the mean sound level of the original field recordings (123.8 dB re 1 µPa), and was within the typical ambient sound level range for oyster reefs sampled in Pamlico Sound.

**Figure 6:**
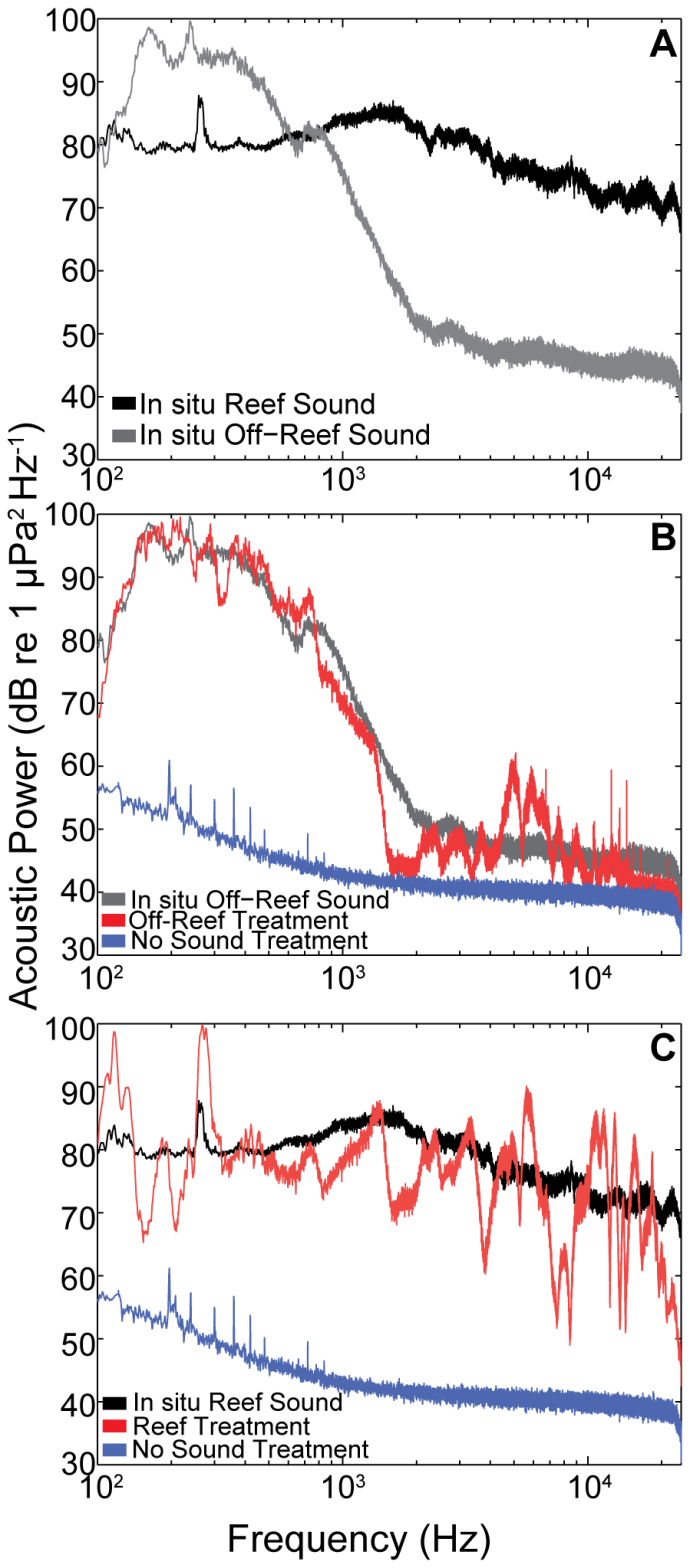
Spectral composition of one set of original and replayed sounds used in lab experiment 2. a) Reef and off-reef sounds recorded simultaneously in West Bay, NC in July 2010. b) Comparison of in situ off-reef sound, replayed off-reef sound in experimental tank and no sound tank spectra. c) Comparison of in situ reef sound, replayed reef sound in experimental tank and no sound tank spectra. Power spectral density estimated via Welch's method (Hamming window, 1-sec averages with 50% overlap).

The reef and off-reef sounds used as treatments in experiment 2 were distinct in their spectral composition (Fig. 6a), with reef recordings composed of higher levels of sound at higher frequencies (> 2 kHz) compared to off-reef recordings, which showed peak levels between 100–500 Hz and a sharp decrease in sound at frequencies above 1 kHz. The power spectra of the sound treatments broadcast by underwater speakers in experimental tanks were similar to the *in situ* recordings in their general pattern (Fig. 6b, 6c). The replayed off-reef sound (Fig. 6b) matched the original recording with more consistency than the reef sound replay (Fig. 6c) for which certain higher frequencies were more difficult to reproduce. Overall, the experimental tank sound treatments differed substantially in terms of frequency composition, with reef sound treatments exposing larvae to the relatively higher sound levels at the higher frequencies measured at our field sites.

For the on reef treatments, broadband rms sound pressure levels ranged between 114.4 and 128.0 dB re 1 µPa and the measured rms acceleration in the 0.1–6 kHz band varied between −45.0 and −37.8 dB re 1 m/s^2^. For the soft bottom treatments, broadband rms sound pressure levels ranged between 109.2 and 120.3 dB re 1 µPa and measured rms acceleration in the 0.1–6 kHz band varied between −50.33 and −37.0 dB re 1 m/s^2^. These replayed sound levels closely matched (within 1–4 dB) the broadband rms sound pressure levels of the field sound recordings.

#### Experiment 1

**Figure 7:**
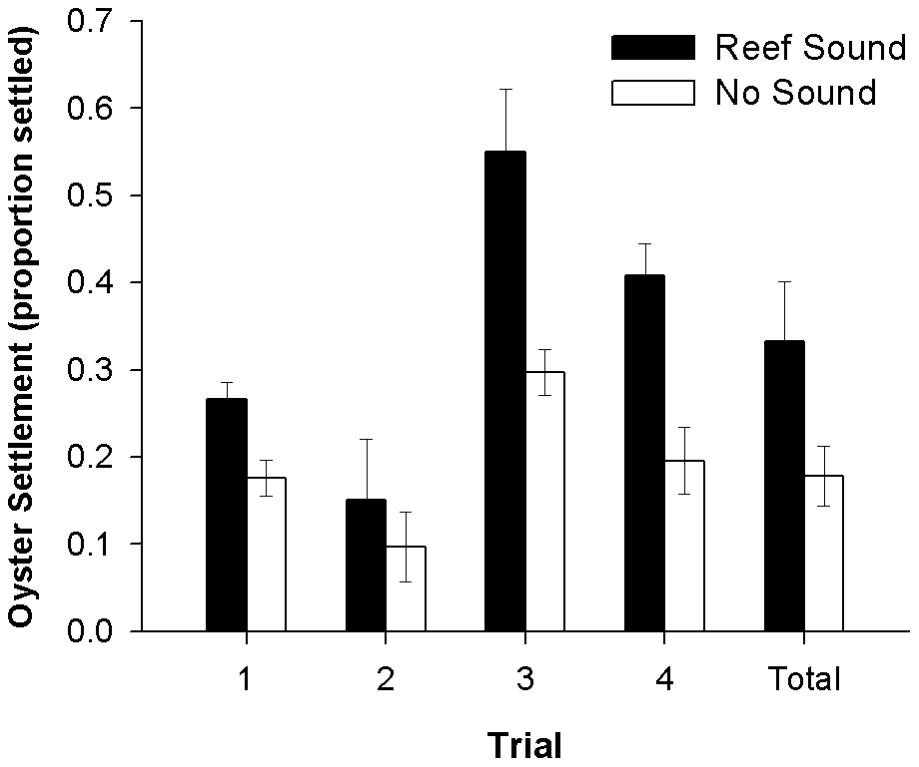
Mean oyster settlement in reef sound and no sound treatments for laboratory experiment 1 trials. Settlement was measured as proportion of larvae settled in a culture at the termination of a trial. Error bars represent 1 S.E. N = 28.

Oyster larval settlement was significantly higher in response to the reef sound treatment compared to no sound (Control) treatment in the 2010 laboratory experiment (Fig. 7; ANOVA: F_1,20_ = 28.75, p<.0001). There was no significant interaction between trial and treatment (F_3,20_ = 2.75, p>.05).

#### Experiment 2

**Figure 8:**
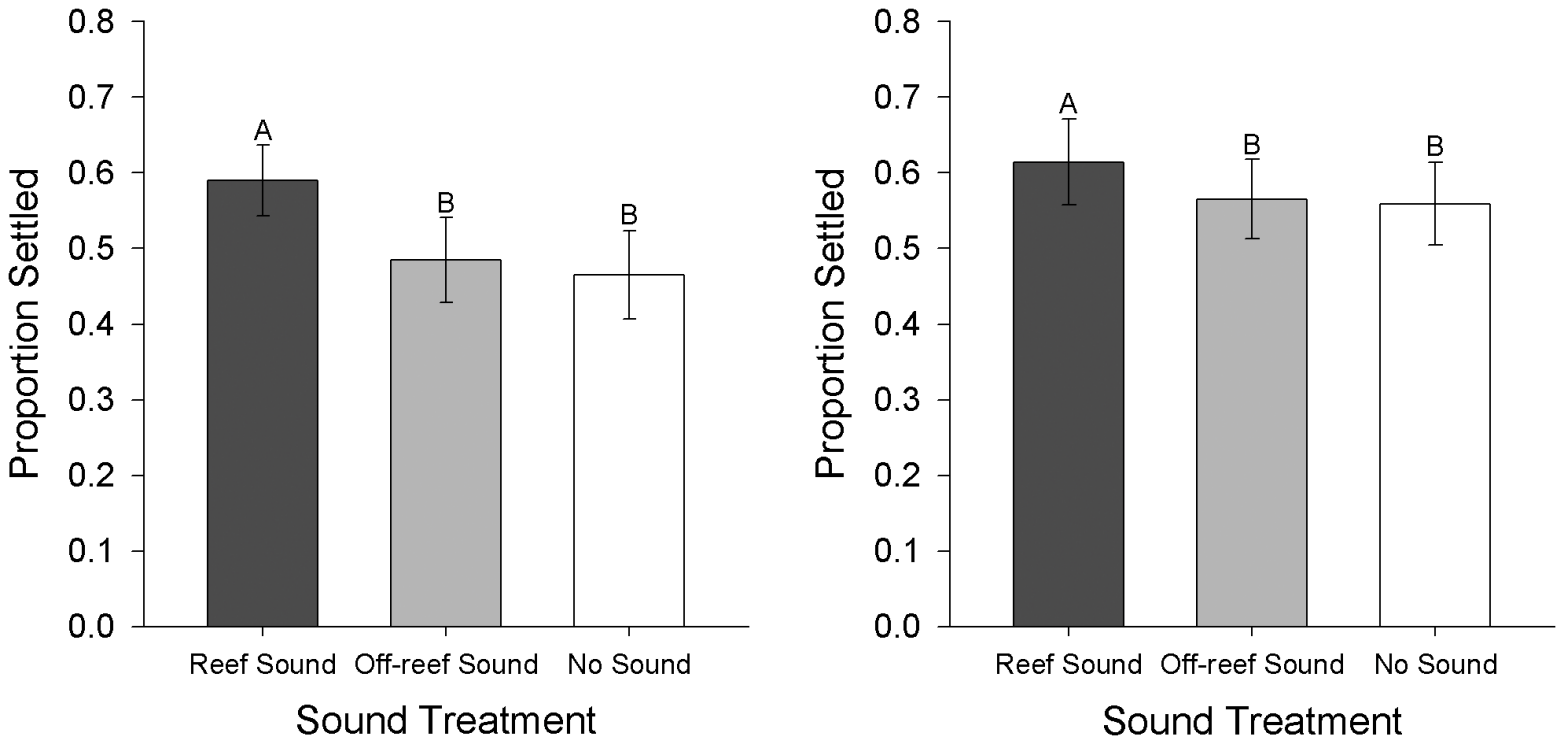
Mean oyster settlement in reef sound, off-reef sound and no sound treatments for experiment 2 trials. with a) no added substrate (N = 15), and b) with oyster shell substrate (N = 27). Letters denote statistically significant differences between treatments based on Tukey's HSD test. Error bars represent 1 S.E.

In the second laboratory experiment, oyster larval settlement was significantly higher when exposed to the reef sound treatment (with and without preferred substrate) compared to both the off-reef sound treatment and no sound treatment (Fig.8; Randomized block ANOVA: Expt. 2a: F_2,8_ = 20.12, p<.001, Expt. 2b: F_2,16_ = 15.59, p<.001). Mean oyster larval settlement for the off-reef sound treatment did not significantly differ from the no sound treatment (Tukey's HSD test, p>.05).

### Larval settlement experiment (Field)

#### Sound treatments

**Figure 9:**
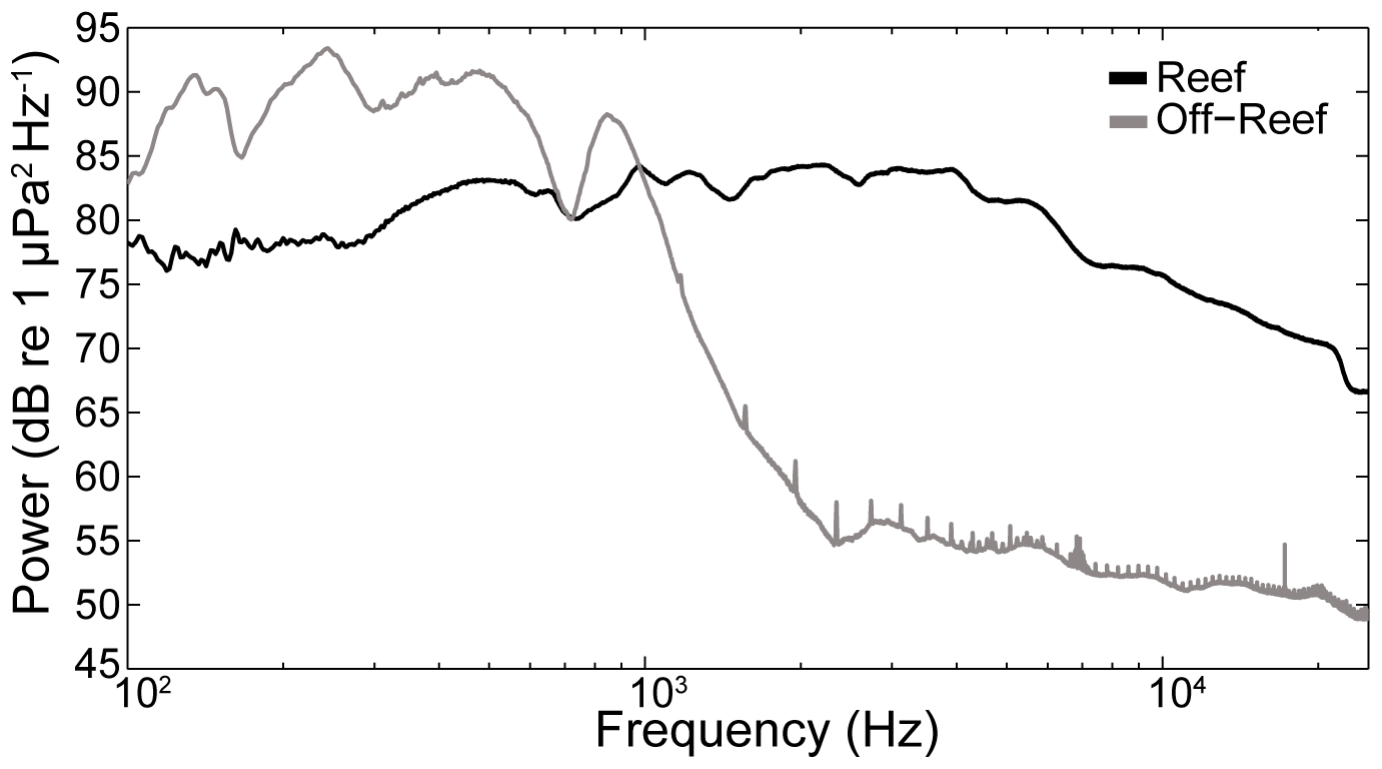
Acoustic analysis of ambient underwater sound at field experiment sites. a) Spectral composition of sound recorded at the reef and off-reef field sites during the field larval settlement experiments in August 2012. Power spectral density estimated via Welch's method (Hamming window, 1-sec averages with 50% overlap). b) Comparison of broadband root-mean square sound levels (in dB re 1 µPa) measured at the reef and off-reef site for the duration of the August 2012 field settlement experiment. The root-mean-square sound level was calculated in a series of non-overlapping 10-sec duration windows.

The sound levels and frequency composition in the field varied between oyster reef and off-reef sites during the August 2012 field experiment (Fig. 9). The mean rms sound level at the reef site (120.06±1.24 dB re 1 µPa) was higher than the more variable off-reef site sound level (114.62±3.55 dB re 1 µPa). Consistent with the habitat-related acoustic measurements described above, the spectral composition of sound at the reef site provided a substantial contribution of higher frequency (1.5–20 kHz) sounds relative to the off-reef site, which was dominated by lower frequency (0.1–1.5 kHz) sounds (Fig. 9). Based on spectral analysis, the elevated sound levels between 100–1000 Hz at the off-reef location were most likely influenced by sciaenid fish species known to be acoustically dominant in NC estuaries [47], [48], while the reef location spectrum consisted of the higher frequency (1.5–20 kHz) invertebrate-generated components.

#### Oyster larval settlement

**Figure 10:**
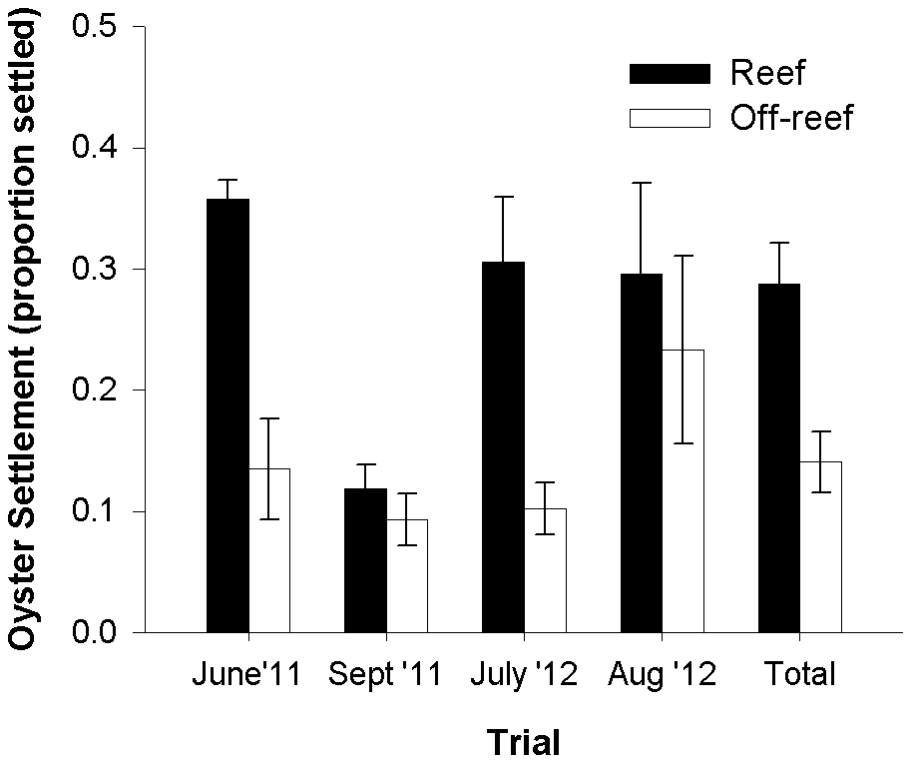
Mean oyster settlement for field-based settlement experimental trials. Settlement was measured as proportion of larvae settled in a culture at the termination of a trial. Error bars represent 1 S.E. N = 32.

In agreement with the laboratory results, oyster larval settlement in larval housings suspended in oyster reef habitats during the field-based experiment was significantly higher compared to larval settlement in off-reef sites (Fig. 10; ANOVA: F_1,24_ = 15.13, p<.001). This overall treatment effect was apparently driven by the June 2011 and July 2012 trials; however, because the statistical model did not find a significant trial by treatment interaction (F_3,24_ = 2.20, p>.05), data were pooled across trials for the overall treatment effect test.

## Discussion

The results of this study provide the first evidence, to our knowledge, that ambient underwater sound associated with adult habitat could be a cue for settlement-stage larvae of an estuarine bivalve mollusk. This has important implications because it is the first study to demonstrate that differences in acoustical characteristics between estuarine habitats have an effect on the larvae of a key ecosystem engineer. Because oyster larval settlement was enhanced in the presence of oyster reef sounds, but not off-reef soft-bottom sounds, it suggests that oyster larval responses are tuned to their preferred settlement environment and that the specific quality and quantity of habitat-related sound is important. The threshold sound levels and component frequencies of oyster reef sound to which oyster larvae respond remain unclear at this initial stage of investigation. An essential extension of this work will be to measure settlement in response to various sound intensity levels relevant to field soundscape measurements and component frequency bands, as has been done to determine the relevant acoustic stimuli for certain fish and crustacean larvae [24], [49].

A number of physical and chemical cues associated with adult habitat, such as rugosity, surface composition, conspecific-produced chemical substances, and bacterial surface films can induce settlement and metamorphosis in larvae of *Crassostrea virginica* [35]. These cues likely act on a small scale (centimeters) to affect habitat selection and induce attachment to the substrate. In contrast, acoustic signals could be useful at a relatively broader spatial scale (meters to kilometers) to facilitate initial orientation to and subsequent encounter with preferred substrate. Given the large distances that larvae can travel from adult spawning habitat to eventual adult oyster settlement habitat (10 s km; [50]), there is clear adaptive value in the use of a reliable signal of appropriate settlement habitat to descend upon, rather than aimless searching of vast swathes of unstructured bottom habitat.

There are several possible mechanistic explanations for the enhanced settlement of oysters in the presence of habitat sounds. While strong swimming larvae such as fish and crustaceans may have the ability to, under certain current flow conditions, navigate by directing their horizontal movement in response to environmental stimuli such as sound [14], [23], weak swimmers such as bivalve veligers are more likely to exhibit “partial navigation” by vertical movement that facilitates transport to settlement habitat [3]. Because habitat-related sounds should reliably indicate close proximity to suitable settlement substrate, elicitation of sinking or downward swimming behavior by these cues could increase the efficiency and success of settlement. Delayed settlement and metamorphosis until encounter of specific settlement cues also represents a mechanism of larval habitat selection that is characteristic of most groups of benthic invertebrates [51]. The increased settlement observed in cultures exposed to oyster reef sounds in this study could represent an alteration of development trajectory or stimulation of physiological changes that promote response to other settlement inducers, as was recently implicated in a study of the response of sea urchin larvae to turbulent shear [52]. Additionally, while sound should be most valuable as a broad-scale cue that facilitates encounter with settlement habitat, habitat sound characteristics could serve an additional habitat selection function if larvae use this information to settle under certain acoustic conditions that reflect high quality habitat. For example, a more productive and higher density oyster reef may be more soniferous than a degraded reef in which settling would be less advantageous.

There are numerous ways that habitat sounds could improve settlement outcomes by providing both early signals to aid larvae in substrate contact, and as an indicator of habitat quality. The elevated sound levels and frequencies of the oyster reef soundscape could trigger both behavioral and physiological changes that expedite the settlement process, thereby increasing the measured settlement in our experimental cultures exposed to reef sound. Given that our experiments were conducted in small containers and with sufficient time for all larvae to be able to encounter settlement substrate without the aid of a cue, it is not surprising that the effect of the sound treatments was modest. Nevertheless, the relatively small effect size does not diminish the possibility that habitat sound plays a significant role in oyster settlement. These findings demonstrate that exposure to sounds of adult habitat influences oyster settlement, and that the underlying mechanism of this sound-mediated response, as well as the ecological relevance, warrants further study. Future laboratory experiments will extend the current work by examining larval behavioral responses (e.g. swimming activity, sinking rate) to habitat sounds, and by investigating the threshold sound levels and frequencies that elicit settlement responses. The effect of artificial rearing conditions on larval responses is an important consideration in the interpretation of experiments using hatchery animals, particularly in light of evidence that reef fish larvae are influenced by previous acoustic experiences [53]. Unfortunately, obtaining sufficient numbers of wild-caught oyster larvae for use in laboratory experiments is unfeasible. To address this and other concerns associated with artificial lab environments, future field replay experiments will aim to elucidate the ecological significance of sound on natural settlement rates for wild larvae.

The experiments presented here expand our understanding of underwater sound as an influence on ecological processes in marine environments, and highlight the need for a better understanding of a variety of topics related to sound-mediated larval behavior and settlement. The specific sensory mechanism for the larval response to acoustic stimuli remains unknown for this species and the other invertebrates that have shown behavioral or physiological responses to sound [25], [30]–[33], [49]. Thus, the study of larval mechanoreceptors represents a key area for future investigation in this field. It is also important to note that for most marine environments, the relevant spatio-temporal scales of acoustic variation have yet to be explored from the perspective of larval dispersal and settlement, and information specific to the soundscape of estuaries, key settlement and nursery habitats for a multitude of species, is extremely limited. In the context of marine conservation, the emerging evidence of the importance of soundscapes in recruitment processes for a variety of species suggests that protection or restoration of habitats that harbor relatively high densities of sound-producing animals (e.g., snapping shrimp, drumming fish, etc.) also may enhance larval replenishment of sound-receptive benthic species. The potential that larval responses to acoustic cues may be a widespread phenomenon both taxonomically and geographically suggests a need to better understand not only the relevant spatio-temporal soundscape patterns and larval responses, but also how alterations to the natural soundscape (e.g. noise pollution) affect settlement and recruitment of key species such as oysters.
