# Correction: Oyster Larvae Settle in Response to Habitat-Associated Underwater Sounds

**DOI:** 10.1371/annotation/b04bc087-808a-4edc-ae2a-08932bff360c

**Published:** 2014-01-06

**Authors:** Ashlee Lillis, David B. Eggleston, DelWayne R. Bohnenstiehl

Two funding organizations and grants were incorrectly omitted from the Funding Statement. The Funding Statement should read: "Funding was provided by National Science Foundation Grants OCE-1234688 and DDIG-1210292, PADI Foundation Grant 5145 and a National Shellfisheries Association Melbourne R. Carriker Student Research Grant. The funders had no role in study design, data collection and analysis, decision to publish, or preparation of the manuscript." 

